# Enhancing neurodivergent student success through wellbeing: Protocol for a quasi-experimental evaluation of the Tuned In Connect program across three Australian universities

**DOI:** 10.1371/journal.pone.0357369

**Published:** 2026-10-01

**Authors:** Genevieve A. Dingle, Rong Han, Radhika Tanksale, Nikki Rickard, Babette Rae, Sonja Pohlman, Dianna Vidas, Martha Munro, Melissa Vallence, Angie Weaver

**Affiliations:** 1 School of Psychology, The University of Queensland, Brisbane, Australia; 2 Centre for Wellbeing Science, Faculty of Education, The University of Melbourne, Melbourne, Australia; 3 School of Psychological Sciences, The University of Newcastle, Newcastle, Australia; 4 School of Psychology, Deakin University, Melbourne, Australia; 5 School of Health and Rehabilitation Sciences, The University of QLD, Brisbane, Australia; PLoS ONE, UNITED STATES OF AMERICA

## Abstract

Neurodivergent university students face significant barriers to participation and success including sensory sensitivities, executive function challenges, social pressures, identity masking, and burnout. Evidence-based programs addressing the social and emotional dimensions of neurodivergent students’ experiences are needed. This protocol describes an evaluation of the Tuned In Connect program, comprising an online self-paced module and a five-session group course. It was adapted by and for neurodivergent university students from the original Tuned In, an emotion regulation intervention that uses a two-dimensional model of emotion and music listening. Participants will be 145 neurodivergent students recruited from three Australian universities. The study will adopt a mixed between- and within-participants design, with participants randomly allocated to the intervention or waitlist control condition, and assessments at baseline and approximately six weeks later. Control participants will have the option of attending the intervention after their 6-week survey and will be asked to complete a third survey after the program. Historical control data from a 2023 cohort of neurodivergent university students may be used to supplement the waitlist control condition. Primary outcomes include sense of belonging, academic motivation, academic course completion, and semester grade point average. Secondary outcomes include emotion regulation, use of music for focus and coping, and wellbeing. Additional secondary and exploratory outcomes include anxiety, burnout, alexithymia and alcohol use. A qualitative sub-study will explore participant experiences through post-program focus groups analysed using reflexive thematic analysis. The findings, if positive, will support the adoption of Tuned In Connect by other universities as a scalable approach to supporting neurodivergent students.

**Trial registration:**

https://www.acses.edu.au/trial/enhancing-success-for-neurodivergent-students-through-wellbeing-a-quasi-experimental-design-study-across-three-universities/.

## Introduction

Neurodivergent individuals, including autistic individuals, individuals with attention deficit hyperactivity disorder (ADHD), learning disabilities, and Tourette’s syndrome, represent a growing proportion of the higher education student population [1]. In Australia, the number of university students disclosing disability or neurocognitive differences has risen steadily, with undergraduate students with disability increasing by 123% between 2008 and 2017 [2]. Universities have responded by expanding academic accommodations such as examination modifications and deadline extensions, and students generally report being satisfied with these [3]. However, a range of other significant barriers to neurodivergent students’ full participation and academic success remain inadequately addressed. These include sensory sensitivities to noise, lighting, and crowded spaces, which can make campus environments overwhelming. Executive function difficulties, including managing transitions between tasks, initiating study, scheduling, and handling academic workload, create persistent obstacles [4]. Social demands, such as group assignments, class discussions, and the pressure to forge peer connections, can be particularly challenging [5]. These academic, sensory, and social demands may also place additional pressure on students’ emotion regulation, particularly when students have difficulty recognising and identifying internal emotional and bodily signals. This may include interoceptive differences, referring to differences in noticing and interpreting internal bodily sensations [6], and alexithymia, referring to difficulties identifying and describing emotions [7]. The cumulative effect of navigating these demands frequently leads to burnout, which involves extreme mental, psychological, academic, and physical exhaustion and can result in disengagement and increased dropout rates [8].

Compounding these difficulties, many neurodivergent students engage in masking, a strategy involving suppressing or concealing their authentic neurodivergent identity and performing neurotypical behaviours to fit in socially [9]. This effortful masking is itself a source of fatigue and psychological distress. It can also involve ‘double masking,’ where students additionally experience a sense of failure when they cannot meet strength-based, neurodiversity-affirming expectations [9,10]. Neurodivergent students may also face anxiety around class participation and stigma related to their neurodivergence, which can negatively affect their relationships with peers and teaching staff [3]. Together, these barriers can influence the quality of students’ experience in higher education, affecting wellbeing and leading to academic failure and dropout [11,12]. While academic accommodations address some of these needs, evidence-based programs co-adapted with neurodivergent students and informed by their lived experiences are needed that go beyond individual academic adjustments. Such programs could help address the social, emotional, and psychological barriers that affect neurodivergent students’ university participation and success.

### Using music for social and emotional wellbeing

Many of the challenges faced by neurodivergent students, including burnout, anxiety, and academic stress, are linked to difficulties in emotion regulation. Emotion regulation can be conceptualized as the ability to modify emotion in flexible and adaptive ways in response to the social context [13]. In neurodivergent populations, emotion regulation may be shaped by differences in emotional awareness, sensory processing, cognitive load, and alexithymia, which can influence how emotions are recognised, experienced, and managed [14–16]. Given its central role in both psychological wellbeing and academic functioning, emotion regulation represents a key target for intervention in this population. Music may be particularly helpful in this context because it can provide a structured and non-verbal way to notice, express, and make sense of emotions, including for individuals who find it difficult to identify or describe their emotional experiences [17,18].

Music listening is associated with wellbeing in young people through several social and emotional pathways [19]. It has been identified as an accessible and effective strategy for supporting emotion regulation [20,21]. In neurodivergent populations, emerging evidence suggests that music may support emotional, social, behavioural, and cognitive functioning, particularly among autistic individuals and people with ADHD [22–24]. There is also evidence that sharing and engaging with self-selected music and live music events can promote feelings of social connectedness [25]. These social effects are particularly relevant for neurodivergent students, who may experience isolation and difficulties with a sense of disconnection from peers and the wider university community. Qualitative and lived-experience research further suggests that autistic individuals may use music in everyday life for emotional self-regulation, sensory support, identity, and social connection [26–29].

The Tuned In program is a music-listening based psychoeducational program designed to help young people recognise and manage their emotions [30]. The program draws on Russell’s circumplex model of emotion, which maps emotions onto the two-dimensional space bounded by energy (high to low) on the vertical axis and valence (negative to positive) on the horizontal axis [31]. Participants learn to identify where their current and desired emotional states sit on these dimensions and to select music that can shift their emotional state in the desired direction. This approach is consistent with the two dimensions used in the Music eScape emotion regulation application [32] and the iso principle in music therapy, in which music is first selected to match a person’s current emotional state and then gradually used to support movement toward a desired emotional state [33]. While music is shared in sessions to explore various emotions, participants engage in one of three activities: imagery drawing, body scanning, and lyric analysis. The activities are informed by Juslin’s BRECVEMA framework [34], which proposes that music influences emotions through multiple pathways, including associations with personal memories, the meaning conveyed by lyrics, and mental imagery triggered by listening.

The original Tuned In program has demonstrated positive effects on emotion regulation in adolescents in school and community settings in Australia [35] and Hong Kong [36] and a four-session Tuned In Uni Students program is effective among university students [30,37,38]. However, the program was developed and evaluated in general student populations and did not account for the distinct cognitive, sensory, and social experiences of neurodivergent students.

### The Tuned In Connect program

To address this gap, the Tuned In Connect program [39] was adapted from the original Tuned In program through an iterative qualitative participatory adaptation process with 11 neurodivergent students during 2024 and 2025. In the first phase, three neurodivergent students completed the original four-session Tuned In program and then took part in post-program interviews. Reflexive thematic analysis of these interviews identified program strengths, including emotional awareness and regulation, engagement, inclusion, and connection, as well as areas requiring neurodiversity-affirming adaptation, including music and emotion content, program delivery, and accessibility. This feedback informed the development of the five-session Tuned In Connect program. In the second phase, Tuned In Connect was delivered to neurodivergent students, with eight students taking part across the two groups. Post-program focus groups were then used to gather feedback on the program’s accessibility, feasibility, neurodiversity-affirming content, group process, and support for emotion regulation. This feedback was used to further refine the program so that it better aligned with neurodivergent students’ lived experiences and support needs.

The program comprises two components designed to support participants to develop a greater understanding of their emotional states and to use intentional music listening as a strategy for emotion regulation. The first is an online, self-paced module [40] that introduces key concepts and strategies from the Tuned In program in an interactive format that takes about 20 minutes to complete. The second is a manualised five-session group course delivered weekly (online or in person) by trained and supervised postgraduate psychology students. The course supports participants to understand the purpose of emotions, identify their current emotional state along the dimensions of energy and comfort, and use music listening as a strategy to regulate emotions across common academic situations. It also normalises and provides strategies for managing academic challenges such as perfectionism, rumination, and procrastination.

Adaptations were made to enhance the relevance and accessibility of the program for neurodivergent students (to be reported in detail elsewhere [41]), and summary of program is in Table 1. One important change included modifying the two-dimensional emotion model by replacing the original valence wording of *positive to negative* emotions with *comfortable to uncomfortable* emotions, in response to student feedback that this wording better captured their emotional experiences. The adaptation also involved revising language, scenarios, and examples across the original four sessions to make the content more accessible and relatable for neurodivergent students. A fifth session was added to address needs identified through student feedback, including recognising and managing burnout through energy ‘accounting’, tolerating uncertainty, taking short breaks to recharge, addressing guilt linked with self-critical thinking, and practising self-compassion. Energy accounting is a neurodiversity-affirming strategy that involves identifying activities that drain or restore energy and adjusting to reduce stress and support wellbeing [42]. Finally, the group format provides a neurodiversity-affirming environment where participants can connect with peers who share similar experiences, with music serving as a shared medium for interaction that may support social connection and reduce feelings of disconnection [43].

**Table 1:** Overview of the Tuned In Connect program sessions.

Session	Title	Focus	Key content	Key adaptation
**1**	Feelings are your friends	Understanding emotions	Purpose of emotions; two-dimensional emotion model; using music to tune into emotions	Neurodiversity-affirming framing and accessible emotion language
**2**	Fearing the worst	Managing anxiety	Anticipatory anxiety; panic; music as a calming tool; procrastination; stress and anxiety	Scenarios and examples adapted to be more relatable for neurodivergent students, with attention to academic anxiety, panic, procrastination, stress, and practical calming strategies
**3**	Listen to yourself	Self-awareness and self-care	Coping with uncertainty; recognising burnout; self-care; self-compassion and music	Added focus on uncertainty, burnout signs, self-care, self-compassion, and using music to support self-compassion
**4**	Staying motivated	Academic motivation	Using music to energise; managing perfectionism; dealing with disappointment; rumination	Scenarios and examples adapted to be more relatable for neurodivergent students, with attention to motivation, perfectionism, disappointment, and rumination
**5**	Tune in to success	Burnout and positive emotions	Managing fatigue and burnout; tuning into positive emotions; energy management strategies	Added session content on fatigue, burnout prevention, positive emotions, energy accounting, and using music as an energy management strategy

### Study objectives and hypotheses

The Tuned In Connect program is designed to improve outcomes for neurodivergent students through two key pathways. First, by teaching participants to recognise and regulate their academic-related emotions using music-based strategies, the program aims to strengthen students’ confidence in managing stress, anxiety, and overwhelm, and to help prevent burnout, which can interfere with academic progress [4,8]. Improvements in emotion regulation are expected to support greater academic engagement and persistence, reflected in outcomes such as course completion and grade point average (GPA). Second, by providing a neurodiversity-affirming group experience in the company of peers who share similar experiences, we expect the program to foster a greater sense of belonging and inclusion at university, which has been identified as an important factor in student retention and success [12]. The shared music listening within the group is also expected to strengthen social connectedness among participants [25,43]. The study will test two hypotheses:

**H1:** From baseline to post-program, compared to controls, students in the intervention condition will show greater improvements in sense of belonging at university and academic motivation, and will have more favourable end-of-semester academic outcomes, indicated by fewer course withdrawals or failures and higher semester GPA.
**H2:** From baseline to post-program, compared to controls, students in the intervention condition will show greater improvements in emotion regulation, use of music for focusing and coping with stress, and wellbeing, and will report lower risky drinking.

Additional secondary and exploratory analyses will examine anxiety, burnout, and alexithymia. In addition, a qualitative study will evaluate the acceptability, relevance, and perceived effectiveness of the program from participants’ perspectives.

## Materials and methods

### Study design

This study employs a quasi-experimental, mixed between- and within-participants design with two conditions: Tuned In Connect (TIC) and waitlist control (WLC), and two time points: baseline and post-program, approximately six weeks later. Participants recruited during the trial period will be randomly allocated to either the TIC or WLC condition. The design is considered quasi-experimental because historical control data collected from neurodivergent students at the University of Queensland in 2023, prior to the development of Tuned In Connect, may also be used to supplement the wait list control condition to increase statistical power and support feasibility. Primary analyses will focus on contemporaneously recruited participants where sufficient data are available, with supplementary analyses including historical controls where appropriate. Potential cohort, site, and timing differences between historical and contemporaneous control participants will be examined and addressed in the statistical analyses.

Ethical approval for this study has been obtained from the Human Research Ethics Committee at The University of Queensland (approval number: HREC 2025/HE001951), with reciprocal arrangements at participating institutions. All participants will provide informed consent prior to participation. Participation may involve psychological discomfort when reflecting on emotions, wellbeing, burnout, or academic challenges, or when taking part in group-based activities. To reduce this risk, groups will be facilitated from a neurodiversity-affirming perspective, and participants will be encouraged to participate in their own style and at their own pace. Participants may skip any questions or withdraw at any time without penalty and will be provided with appropriate support resources if needed.

### Participants and recruitment

Participants will be recruited from three Australian universities: the University of Queensland, the University of Newcastle, and the University of Melbourne. These universities were selected to support multi-site implementation across different institutional contexts, including variation in student support structures, neurodiversity-related services, campus settings, and student equity profiles. For instance, the University of Queensland and the University of Melbourne have more established neurodiversity hubs than the University of Newcastle. Also, the University of Newcastle has a high proportion of low socioeconomic status and “first in family” students and most of their students work in addition to studying. Including three universities allows the study to examine whether Tuned In Connect can be delivered beyond a single institution and whether recruitment, engagement, and outcomes are feasible across different university settings. The study will be promoted through university disability and student support services, campus communications, social media, and word of mouth. Recruitment commenced in January 2026 and will continue until 30 September (or earlier if the target sample size is reached), data collection commenced on 26 February 2026 and is expected to be completed by 24 December 2026 and results available by 28 February 2027.

Eligible participants are students currently enrolled at one of the participating universities who either hold a formal neurodivergent diagnosis (including but not limited to autism, ADHD, learning disability, or Tourette’s syndrome) or self-identify as neurodivergent. Self-identification has been included as an eligibility pathway in recognition of the significant cost and lengthy waiting times associated with formal assessment and diagnosis, alignment with access requirements for university support services, and respect for student autonomy and diverse neurodivergent experiences [44,45]. Participants may be domestic or international students of any age, gender, and ethnicity, enrolled in any faculty or discipline at the undergraduate or postgraduate level. Administrative academic outcome data, including course completion, withdrawal, failure, and semester GPA, will be retrieved only for participants who provide consent and where these outcomes are applicable to their enrolment type. For example, GPA may not be available for research-only postgraduate students, who will remain eligible for the study but will be excluded from analyses of GPA-based outcomes where these data are not applicable. The number of participants with available administrative academic data will be reported. Participants will receive reimbursement of up to AUD $60 for completing study assessments, with partial reimbursement provided according to the number of surveys completed, to support recruitment and minimise attrition. Participants who take part in focus groups will receive an additional AUD $20 voucher.

### Sample size

An *a priori* power analysis was conducted using G*Power [46] to determine the required sample size for the primary analyses. Based on a medium interaction effect size, statistical power of.80, and a Bonferroni-adjusted alpha level of.00625 to account for multiple planned outcome tests, the required sample size is estimated at *N* = 145. The study will therefore aim to recruit 145 neurodivergent students across the three universities, subject to recruitment feasibility. Newly recruited participants will be randomly allocated to the Tuned In Connect intervention or waitlist control condition. Historical control data from neurodivergent students at the University of Queensland are also available and may be used in supplementary analyses where appropriate, following checks for baseline equivalence with contemporaneously recruited controls.

### Intervention

#### The Tuned In Connect program.

Participants allocated to the intervention condition will receive the full Tuned In Connect package, consisting of an online, self-paced module (approximately 20 minutes) introducing key concepts and strategies, followed by a manualised five-session group program delivered weekly (online or in person). Facilitators will complete standardised training using the program facilitator manual and training videos, attend a live information session with the program lead author, and receive weekly supervision from designated site supervisors as part of their supervised practice placement. Supervision will be coordinated across sites to support consistency in program delivery. Program fidelity will be monitored using session checklists completed after each session (S2 File)

#### Waitlist control (WLC) condition.

Participants allocated to the WLC condition will be asked to complete the same assessment measures at baseline and at the first follow-up, approximately six weeks later. During this time, participants may continue to access any usual supports or services available to them but will not be provided with access to the Tuned In Connect online module and group program. Following completion of the first follow-up assessment, participants in the WLC condition will be offered the opportunity to participate in the intervention. For WLC participants who take up this offer, an additional post-intervention assessment will be administered to them to capture post-intervention outcomes. The timing of this assessment may vary depending on group scheduling and participant availability.

### Procedure

Participants who express interest in the study will be contacted by the project coordinator or trained site research assistants and asked to complete eligibility screening and informed consent. Eligible participants will then complete the baseline survey. Following baseline assessment, participants’ ID codes will be passed on to an independent member of the research team, who will use a computer-generated randomisation sequence to allocate participants to either the intervention or WLC condition while remaining blind to participants’ identities. Randomisation will be monitored across sites to support balance between conditions. Participants allocated to the intervention condition will complete the online self-paced module and attend the five-session group program over approximately six weeks. All participants will complete the first follow-up assessments approximately six weeks after baseline, or as close to this timing as feasible given group scheduling and participant availability.

### Outcomes and measures

#### Primary outcome measures.

**Belonging and inclusion:** Belonging and inclusion will be assessed using two items based on the single item social identity scale [47]: “I feel a sense of belonging as a student at the university” and “I feel included at the university.” Participants will respond on a 5-point Likert scale (1 = strongly disagree, 5 = strongly agree), with higher scores indicating greater perceived belonging and inclusion. A copy of the survey is in S1 File.

**Academic motivation:** Academic motivation will be assessed using a four-item measure used in previous research [37]. The scale assesses attitudes towards academic responsibility, intellectual capacity, engagement, and independent motivation, for example, “I am an involved and engaged student”. Participants will respond on a 5-point Likert scale (1 = strongly disagree, 5 = strongly agree). Items are averaged to produce a score on the measure. Internal reliability analyses will be reported.

**Academic participation and success:** Academic participation and success will be assessed using administrative data, including percentage of students completing the semester, number of course withdrawals and failures, and semester GPAs. These data will be obtained via data linkage for participants who provide consent and will be used as objective indicators of academic outcomes.

#### Secondary outcome measures.

**Emotion regulation:** Emotion regulation will be assessed using five purpose-written items used in previous research [30,35,38], assessing recognition of emotions, ability to name emotions, confidence in regulating emotions, use of music to regulate emotions, and use of other strategies. Participants will respond on a 5-point scale (1 = never true, 5 = almost always true), with higher scores indicating greater emotion regulation capacity. The five items will be averaged to create a composite emotion regulation score, with internal reliability reported.

**Use of music for focus and coping:** Use of music will be assessed using four items adapted from previous studies [21,48], asking participants to nominate a specific piece of music or sound that helps them focus on study and manage stress, and to describe how it supports these functions. These items include both structured and open-ended responses.

**Mental wellbeing:** Mental wellbeing will be assessed using the Short Warwick-Edinburgh Mental Wellbeing Scale (SWEMWBS) [49], a 7-item measure of positive mental health. Items such as: “I’ve been feeling useful” are rated on a 5-point scale (1 = none of the time, 5 = all of the time) and summed to produce a total score in the range of 7–35. Published mean scores are around 23 (*SD* = 4), with higher scores representing better psychological wellbeing. Internal reliability analyses will be reported.

**Alcohol use:** Alcohol use will be assessed using the 3-item Alcohol Use Disorders Identification Test-Consumption (AUDIT-C) [50], which screens for risky drinking. Items are rated on a scale of 0–4 and summed to produce a total score in the range of 0–12. A cutoff of 3 or more for females and 4 or more for males has been suggested to indicate hazardous drinking. Additional items assessing social tension reduction drinking motives [51] will also be included. Participants respond using frequency-based response options, with higher scores indicating greater risk.

#### Additional secondary and exploratory outcome measures.

**Burnout:** Burnout will be assessed using the Autistic Burnout Inventory [52], which measures experiences of exhaustion, reduced capacity, and sensory sensitivity. Participants will respond to items on a 6-point scale (1 = strongly disagree, 6 = strongly agree), with items 3 and 4 reverse coded. The item scores are summed to a total in the range of 8–48, with the published mean for autistic individuals (*M* = 31.37) higher than the mean for non-autistic individuals (*M* = 27.11). Internal reliability analyses will be reported.

**Anxiety:** Anxiety will be assessed using the 4-item subscale from the PsyCheck mental health screener [53], which asks respondents if, over the past 30 days, they have experienced particular symptoms, such as “Do you feel nervous?” They answer 0 = no, 1 = yes, and scores are summed to a total of 0–4. This measure has been used in previous university student studies [37].

**Alexithymia:** Alexithymia will be assessed using the 6-item Perth Alexithymia Questionnaire-Short Form (PAQ-S) [54], which measures difficulties in identifying and describing emotions. Participants respond to items including “When I’m feeling bad, I can’t tell whether I’m sad, angry, or scared” on a 7-point scale (1 = strongly disagree to 7 = strongly agree), with higher scores indicating higher levels of alexithymia. Published means on the measure are around 18 (*SD* = 7). Internal reliability analyses will be reported.

#### Implementation measures.

Process measures will assess participant engagement, program acceptability, and intervention fidelity. Participant experience will be assessed using session ratings collected at the end of each group session. These ratings are based on the Session Rating Scale (SRS) [55], and include four items evaluating relationships, relevance of goals and topics, approach, and overall session experience. Participants will complete these ratings at the end of each session to provide ongoing feedback on program delivery.

Program acceptability will be assessed using program evaluation items administered following completion of the intervention. These items assess perceived helpfulness, engagement, relevance, and likelihood of recommending the program to others. Participants will respond on a percentage scale (0% = not at all, 100% = absolutely). For participants in the WLC condition, these items will be administered following their delayed participation in the intervention. Intervention fidelity will be assessed using facilitator-completed session checklists at the end of each group session, capturing whether key components were delivered as intended. Fidelity will be further supported through standardised facilitator training and ongoing supervision to ensure consistency across facilitators and sites.

Qualitative data will be collected from a subset of approximately 20 intervention participants via semi-structured focus groups or individual interviews conducted after completion of the program. All intervention participants who complete the program will be invited to take part in the qualitative sub-study, including through an invitation at the end of the final group session. Participation will be optional. Students who are interested and available may remain after the final session or arrange a mutually suitable time to take part. The researchers will aim to include participants from different university sites and delivery modes where possible. Participants will be invited to provide feedback on the acceptability, relevance, and perceived impact of the program, including aspects they found helpful, areas for improvement, accessibility of content, session structure, and perceived support for neurodivergent students. Focus groups or interviews will be conducted online at a mutually suitable time and are expected to take less than 30 minutes. They will follow a semi-structured interview guide developed by the research team. Discussions will be audio-recorded and transcribed for analysis. The full interview questions are provided in S3 File.

### Data analysis plan

All survey data will be collected electronically using Qualtrics software. A backend coding system will link participants’ survey responses across time points without requiring identifiable information for survey linkage. With participant consent, academic outcome data will be retrieved from university records at the end of the current semester only.

Quantitative analyses will examine changes in primary and secondary outcomes over time between conditions. The primary analyses will use mixed-effects models, with model type selected according to the distribution of each outcome. Continuous outcomes will be analysed using linear mixed-effects models, and binary or count academic outcomes will be analysed using appropriate generalised mixed-effects models. For each outcome, models will include fixed effects of condition, time, and the condition by time interaction, with a random intercept for participant. The condition by time interaction will be the primary test of intervention effects. This approach allows participants with incomplete outcome data to be retained in the analysis where appropriate and is flexible for handling missing data and unequal group sizes. Variation in follow-up timing will be examined and, if needed, included as a covariate or addressed in sensitivity analyses.

Baseline equivalence between conditions will be examined for demographic, enrolment, site, delivery mode, and control-source variables, including age, gender, domestic or international enrolment status, undergraduate or postgraduate enrolment, university site, neurodivergent diagnosis or self-identification status, and whether control data are historical or contemporaneously recruited. Variables that differ meaningfully between conditions and are theoretically relevant to the outcomes will be included as covariates where appropriate. Baseline scores for the outcome being analysed will not be entered as additional covariates in the repeated-measures mixed-effects models, as these scores are already included as part of the time factor.

A Bonferroni-adjusted significance threshold will be applied to the planned primary and key secondary outcome tests: belonging and inclusion, academic motivation, course withdrawal or failure, semester GPA, emotion regulation, use of music for focus and coping, wellbeing, and alcohol use. Based on eight planned outcome tests, the adjusted alpha will be α = .00625 (.05/8). Effect sizes and confidence intervals will be reported. Additional secondary outcomes, including burnout, anxiety, and alexithymia, will be analysed using the same mixed-effects modelling approach where appropriate. These outcomes will be interpreted as exploratory. Implementation measures, including session ratings, program acceptability, and facilitator fidelity checklists, will be summarised descriptively. For the qualitative data, transcripts will be analysed using reflexive thematic analysis [56].

## Discussion

This study represents the first evaluation of Tuned In Connect, a neurodivergent-informed adaptation of the Tuned In program, designed to address the emotional, sensory, and academic challenges experienced by neurodivergent university students. This represents a key innovation in extending an existing program to better meet the social and emotional needs of neurodivergent students in higher education. The program incorporates modifications informed by consultation with neurodivergent students during the adaptation process, including reframing emotional dimensions, addressing burnout and uncertainty, and integrating self-compassion strategies. It also emphasises the use of self-selected music as a flexible and personalised tool for emotion regulation, allowing participants to engage with strategies that align with their sensory preferences and everyday experiences. These changes reflect growing recognition that interventions need to be tailored to the cognitive, sensory, and social experiences of neurodivergent individuals to be effective [10]. The inclusion of a group-based format further enables peer connection in a neurodiversity-affirming environment, which may support belonging and reduce isolation, both of which are critical for student retention [12].

Another important strength of the study is the integration of self-report measures and objective academic indicators. In addition to self-reported measures of wellbeing and emotion regulation, the study includes academic outcomes such as course completion and grade point average, providing a more comprehensive assessment of intervention impact. This approach addresses a limitation in existing research, which has often relied on psychological outcomes alone. The study also has an accessible delivery model, as the program can be delivered either in person or online. This allows participants to choose the format that best fits their access needs, sensory preferences, location, and availability, and may support implementation across different university contexts.

Several limitations should be acknowledged. The quasi-experimental design may introduce selection bias and limit causal inference. In addition, the relatively short follow-up period limits conclusions regarding the longer-term sustainability of effects. Despite these limitations, the study has important implications for the development of preventive mental health supports in higher education. The multi-site implementation across three universities suggests that the program can be delivered across diverse university contexts, supporting its potential scalability and applicability within existing student support systems. By combining a low-intensity, scalable format with strategies that can be applied in everyday contexts, the program has the potential to complement existing university services and reach students who may not engage with traditional supports. The use of music listening as an emotion regulation strategy may further enhance accessibility and engagement, particularly for students who prefer non-clinical approaches to managing their mental health. Music may also be a particularly meaningful and engaging medium for many neurodivergent students, as it can provide a flexible, personalised, and non-verbal way to notice, express, and regulate emotions.

Future research may build on this work by examining longer-term outcomes, testing the program using fully randomised designs, and exploring adaptations for different student populations and delivery formats. Further participatory adaptation or co-design work with a more diverse range of neurodivergent students would also be valuable, particularly to understand how preferences and support needs may differ across neurodivergent identities, study levels, cultural backgrounds, and delivery contexts. Further investigation into the mechanisms of change, particularly the role of emotion regulation and social connection, may also help refine and strengthen the intervention.

In conclusion, this study evaluates a neurodivergent-specific intervention that uses music-based emotion regulation strategies to support psychological and academic outcomes. The findings will contribute to the growing evidence base for accessible and preventive mental health interventions in higher education and inform the development of programs that better support the diverse needs of university students.

## Supporting information

S1 File Survey instruments. Complete baseline and post-assessment survey items including demographic questions, primary and secondary outcome measures, and program evaluation items.
(PDF)

S2 File Session fidelity checklist. Checklist for group facilitators to complete at the end of each session to monitor program delivery quality and consistency.
(PDF)

S3 File Semi-structured interview schedule. Interview guide for post-program focus groups and individual interviews with Tuned In Connect intervention participants.
(PDF)
