# Supplementary material for: Enhancing neurodivergent student success through wellbeing: Protocol for a quasi-experimental evaluation of the Tuned In Connect program across three Australian universities
Source: PLoS One. 2026 Oct 1;21(10):e0357369. doi: 10.1371/journal.pone.0357369 (PMC13630211; doi:10.1371/journal.pone.0357369)
Supplement: Supplementary file 1 [file pone.0357369.s001.pdf]

Enhancing Neurodivergent Student Success through Wellbeing: Study across Three Universities

(T1 survey only) Part A. About you

1. What is your age (in years, at your last birthday) _____
2. What is your gender?
    a. Female
    b. Male
    c. Non-binary
    d. Prefer not to say
3. What is your nationality? ______________________
4. Do you identify as any of the following (tick all that apply)
    a. Aboriginal, Australian South Sea Islander or Torres Strait Islander
    b. Culturally diverse background
    c. Speaker of language(s) other than English (LOTE)
5. Which of the following conditions do you experience (tick all that apply):
    a. ADHD
    b. Autism / ASD
    c. Learning disorder
    d. Tourette's
    e. Other _______________________
6. In relation to the condition/s you selected above, are these:
    a. Self-identified
    b. In the process of being formally assessed
    c. Formally diagnosed
    d. Some diagnosed, some self-identified

Part B. Being a student

7. Which of these universities are you enrolled in?
    a. University of QLD
    b. University of Newcastle
    c. University of Melbourne
8. Are you enrolled as an
    a. Domestic (Australian) student
    b. International student
9. What level of degree are you enrolled in
    a. Undergraduate including honours
    b. Postgraduate / higher degree

Most questions below are on 5-point ratings from SD=strong disagree, D=disagree, NAD=neither agree nor disagree, A=agree, SA=strongly agree

10. I feel a sense of belonging as a student at the [insert name of University]

11. I feel included at the [insert name of the University]

Academic Motivation

1. I take my own academic responsibilities seriously

2. I believe in my own intellectual capacity
3. I am an involved and engaged student
4. I am an independent, motivated and ambitious learner

Part C. Your music
How frequently do you listen to music while studying? (5-point Likert scale, 1 = Never, 5 = Always)

What do you usually listen to while studying? (tick any that are true)

- chosen music (favourite music, e.g. rock or pop)
- chosen music (music chosen for studying)
- ambient noise
- curated playlist e.g. Spotify study playlist
- radio
- podcasts
- other
- I do not listen to music while studying (branch logic to skip next questions)

Please write the name of a piece of music and artist that you often listen to while studying:

Please explain how or why this music helps you to study: (Open)

Please write the name of a piece of music and artist that you find helps manage your stress:

Please explain how or why this music helps you to manage your stress: (Open)

Part D. Your feelings.
Emotions. Please rate how often these statements were true of you <u>over the past week</u>. (Rating: Never true, Rarely true, Sometimes true, Often true, Almost always true)

1. I was aware of what happened in my body when I felt strong emotions
2. I was able to name these feelings (e.g., I'm happy, sad, nervous, etc)
3. I felt confident that I could manage my strong emotions
4. I use music as a way of managing my emotions
5. I have a range of healthy ways of managing my emotions

Burnout. Please rate each of the following statements according to how you have felt <u>in the past month</u>: (on a 6-point scale from 1= SD, 2=D, 3=SD, 4=SA, 5=A, 6=SA)

1. It became very draining to "act appropriately" or "blend in" when I was with other people.
2. I was much more easily bothered by everyday sounds than I used to be.

3^R. I almost always felt like I had the energy to do what I needed to get done.
4^R. I was able to handle most everyday sensory environments without much trouble.

5. Simple everyday tasks were physically or emotionally draining
6. I felt burned out from everything that I needed to do.
7. I couldn't take care of my responsibilities as much as before.
8. It felt like I couldn't pay attention or stay on task as well as I could in the past.

Anxiety. Please tick any of the following symptoms you have experienced in the past 30 days (yes / no)

- Are you easily frightened?
- Do your hands shake?
- Do you feel nervous?
- Do you have trouble thinking clearly?

Alexithymia. This questionnaire asks about how you perceive and experience your emotions. Please score the following statements according to how much you agree or disagree that the statement is true of you. Circle one answer for each statement.

Some questions mention *bad* or *unpleasant* emotions, this means emotions like sadness, anger, or fear. Some questions mention *good* or *pleasant* emotions, this means emotions like happiness, amusement, or excitement.

		Strongly disagree	----	----	Neither agree nor disagree	----	----	Strongly agree
1	When I'm feeling *bad* (feeling an unpleasant emotion), I can't find the right words to describe those feelings.	1	2	3	4	5	6	7
2	When I'm feeling *bad*, I can't tell whether I'm sad, angry, or scared.	1	2	3	4	5	6	7
3	I tend to ignore how I feel.	1	2	3	4	5	6	7
4	When I'm feeling *good* (feeling a pleasant emotion), I can't find the right words to describe those feelings.	1	2	3	4	5	6	7
5	When I'm feeling *good*, I can't tell whether I'm happy, excited, or amused.	1	2	3	4	5	6	7
6	I don't pay attention to my emotions.	1	2	3	4	5	6	7

Part E. Your health and wellbeing

Wellbeing. Below are some statements about feelings and thoughts. Please tick the box that best describes your experience of each over the last 2 weeks. (1=None of the time, 2=rarely, 3=sometimes, 4=often, 5=All of the time)

1. I've been feeling optimistic about the future
2. I've been feeling useful
3. I've been feeling relaxed
4. I've been dealing with problems well
5. I've been thinking clearly

6. I've been feeling close to other people
7. **7.** I've been able to make up my own mind about things

AUDIT-C. Please tick one box for each question, as it applies to your drinking over <u>the past year</u>:

1. How often do you have a drink containing alcohol ? Never, Monthly or less, 2-4 times/month, 2-3 times/week, 4+ times/week
2. How many standard drinks do you drink on a typical drinking day? 1-2, 3-4, 5-6. 7-9, 10+
3. How often do you have 5 or more drinks on an occasion? Never, Less than monthly, Monthly, Weekly, Daily or almost daily

YAAMS. Listed below are reasons people might be inclined to drink alcohol. Please rate how often each of the following is a reason for drinking on the scale: 1 = almost never/never, 2 = some of the time, 3 = half of the time, 4 = most of the time, 5 = almost always/always
You drink….
…because it helps you feel less afraid that others will not approve of you
…because it helps you worry less about what other people think of you
…to feel more comfortable with others
…to feel less nervous with strangers

(T1 and T2 survey) Consent to link with your academic outcomes for this semester
I give permission for the named researchers to retrieve the number of courses I enrolled in, number of courses completed, and semester GPA from my University records for <u>the current semester only</u>.

☐ Yes. Please give your student number here ________________________

☐ No

(End of each group session). Session rating scale

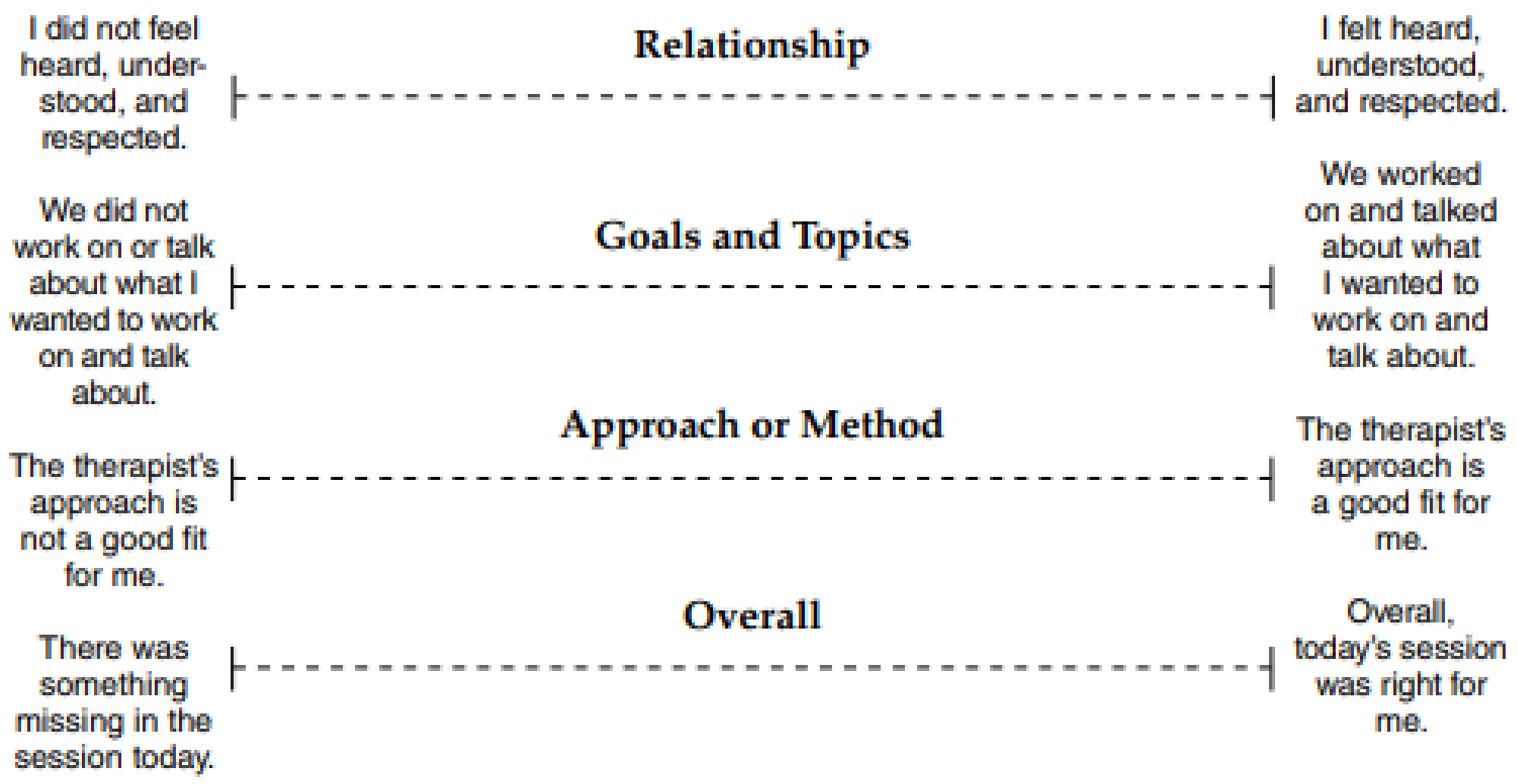

(T2 survey for Intervention, T3 survey for Wait listed participants) Program evaluation

Please rate the Tuned In Connect program on the following, using a scale from 0% = not at all to 100% = absolutely.

1. I found the program helpful
2. I found the program to be engaging
3. I found the scenarios and strategies relevant
4. I would recommend this program to a friend

Any further feedback on what you liked or thought could be done better? (open text box)
