# Supplementary material for: Enhancing neurodivergent student success through wellbeing: Protocol for a quasi-experimental evaluation of the Tuned In Connect program across three Australian universities
Source: PLoS One. 2026 Oct 1;21(10):e0357369. doi: 10.1371/journal.pone.0357369 (PMC13630211; doi:10.1371/journal.pone.0357369)
Supplement: Supplementary file 2 [file pone.0357369.s002.pdf]

Site: ___________ Facilitators names: _____________ Dates of program: __________ Group size (at start): ________ Online / In person (circle one)

Session	Topics	Completed	Notes
1. Feelings are your Friends	• Overview and purpose of the program<br>• Neurodiversity affirming framework<br>• Exploring our relationship with music<br>• Group guidelines<br>• What are emotions and their purpose?<br>• The two-dimensional model of emotions<br>• Music and emotions	☐<br>☐<br>☐<br>☐<br>☐<br>☐<br>☐	
2. Fearing the worst	• Recap and Tune in<br>• Anticipatory anxiety<br>• Panic during a feared event<br>• Music to help calm us<br>• Procrastination<br>• Stress and anxiety	☐<br>☐<br>☐<br>☐<br>☐<br>☐	
3. Listen to yourself	• Recap and Tune in<br>• Breathing Pause<br>• Progress Tracker<br>• Tune in to your current feelings<br>• Coping with uncertainty<br>• Recognising burnout<br>• Self-care and self-compassion	☐<br>☐<br>☐<br>☐<br>☐<br>☐<br>☐	
4. Staying motivated	• Recap and Tune In<br>• Energising to keep studying when you're fatigued.<br>• Perfectionism<br>• Overcoming rumination	☐<br>☐<br>☐<br>☐<br>☐	
5. Tune into success	• Recap and Tune In<br>• Fatigue<br>• Tuning In to positive emotions<br>• Energy Accounting<br><br>Debrief and post-program survey	☐<br>☐<br>☐<br>☐<br><br>☐	
