# Supplementary material for: Enhancing neurodivergent student success through wellbeing: Protocol for a quasi-experimental evaluation of the Tuned In Connect program across three Australian universities
Source: PLoS One. 2026 Oct 1;21(10):e0357369. doi: 10.1371/journal.pone.0357369 (PMC13630211; doi:10.1371/journal.pone.0357369)
Supplement: Supplementary file 3 [file pone.0357369.s003.pdf]

Tuned In Connect – Post-Program Focus Groups and Individual Interviews

Interviewer Preamble

Introduce yourself and thank participants for attending. Ask participants to introduce themselves briefly and indicate which university they are from (if they do not already know each other). Explain that the purpose of the focus group or interview is to give participants a voice in sharing whether the program met their needs, and whether there are any aspects that could be improved for neurodivergent students.

Interview Questions

- What attracted you to participating in the TIC program? [Prompt: For example, was it to help you connect with others, or something about the music, or something else?] (this is trying to better understand their needs – was it related to wanting to feel connection/belonging, or was it curiosity about how music can be used in their studies etc.)
- Do you have any feedback on the program's recruitment process (e.g., thoughts on the flyers, email correspondence etc.)

We're interested in your feedback on the Tuned In Connect program – could you comment first on the things you found helpful or positive about the program?

- Did the program help you notice or manage your emotions in academic settings?
  - (if haven't mentioned yet, prompt) Can you share an example?
- Which parts of the program felt most (or least) relevant to you as a student, and why? Were there any parts that felt less relevant?
- How, if at all, did the program affect how you approach your studies and academic challenges?

Now could you comment on things you found unhelpful or that could be improved about the program?

- Were there any aspects of the program content or program delivery that felt neuro-normative or inaccessible to you?
  - *Prompt: For example, were the materials and communication styles used in the program a good fit for how you think and learn?*
- How did the structure of the sessions (e.g., timing, pace, activities) work for you?
- How supportive do you think the program is for neurodivergent students? Were there any barriers to you participating fully in this program? If yes, what were they?
- As a contributing co-designer of this program with valuable lived experience of neurodivergence, what would you like to see added or changed in future versions of TIC?

Wrap up: Thank participants for their time. Before closing, ask: Are there any important points about your experience that we haven't talked about?

Notes for Interviewers

General guidance:

- Focus groups and individual interviews will be conducted online at a mutually convenient time.
- All sessions will be audio-recorded and transcribed for thematic analysis.
- This guide is semi-structured; interviewers should follow the participant’s lead and use prompts flexibly.
- Approximately 20 intervention participants will be invited to participate.
